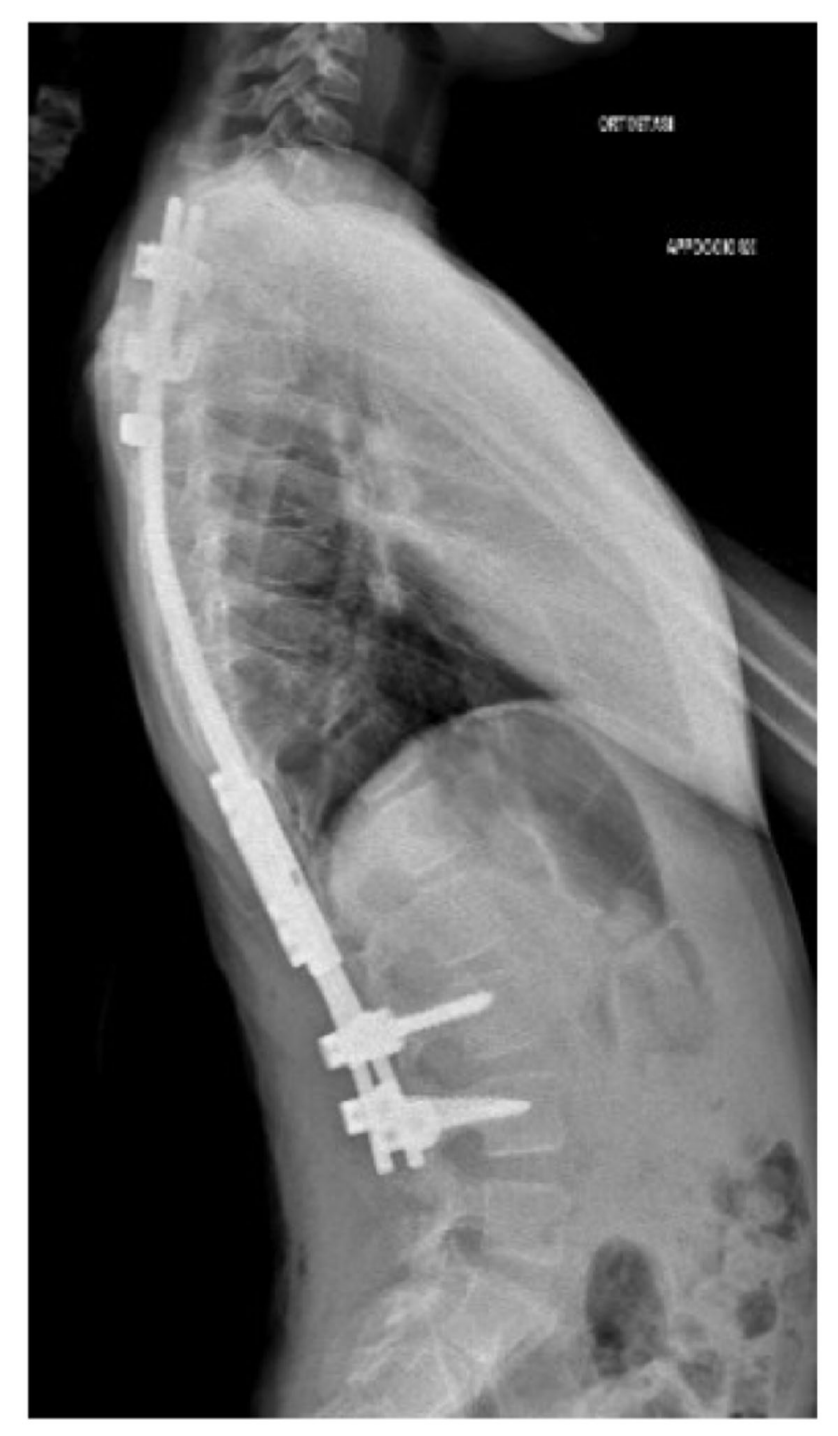# Growing spinal systems and early onset deformities: is hyperkhyphosis a contraindication?

**DOI:** 10.1186/1748-7161-10-S1-O74

**Published:** 2015-01-19

**Authors:** Stefano Giacomini, Mario Di Silvestre, Francesco Lolli, Francesco Vommaro, Konstantinos Martikos, Elena Maredi, Andrea Baioni, Tiziana Greggi

**Affiliations:** 1 Deformities of Spine Surgery, Rizzoli Orthopaedic Institute, Bologna, Italy

## Background

Growing spinal systems are actually used for the treatment of early onset scoliosis. However, they are distraction based systems, so the hyperkyphosis is not considered as a correct indication. Aim of our study is to show if those systems can be effectively used in the treatment of spinal kyphotic deformities.

## Materials and methods

We retrospectively reviewed 16 paediatric patients affected by kyphotic spinal deformity (T3-T12 kyphosys > 60°) surgically treated with Growing Rod or VEPTR-like systems from 2006 to 2011. There were 8 males and 8 females, with a mean age of 7 years (range, 4 to 11). The aetiology was: idiopathic scoliosis (5 cases), kyphosis in Morquio disease (1) and in Pott disease (1), congenital scoliosis (3), trisomy 8 (1), Escobar syndrome (1), Prader Willi (1), spondylocostal dysplasia (1), arthrogryposis (2). Dual growing rod was implanted in 9 cases, VEPTR in 9 (always rib to spine construct).

Pre-operative main thoracic scoliosis averaged 64° (range, 10° to 100°), lumbar scoliosis 55° and thoracic kyphosis 71° (60° to 90°), 67° in patients treated with growing rod and 77° for those treated with VEPTR.

## Results

Mean follow-up was 30 months (range, 18 to 67). After the first surgery, thoracic kyphosis was corrected from a mean value of 71° (range, 60° to 90°) to 52° (21° to 80°) (p<0.05); in cases treated with growing rod, kyphosis was corrected from 67° to 44° (p<0.05), in cases treated with VEPTR from 77° to 60° (p<0.05). At final follow up, after 31 lengthening procedures, a loss of correction occurred on sagittal plane: thoracic kyphosis increased from 52° to 59° (p<0.05); in case of growing rod, from 44° to 50° (p<0.05), in case of VEPTR from 58° to 68° (p<0.05). 15 minor complications occurred in 7 patients, requiring revision surgery in 7.

## Conclusions

Our results showed that growing spinal implants can be safely used in the treatment of kyphotic deformities. Due to distraction procedures, a loss of correction on sagittal plane is commonly observed at follow up. The final result is mostly related to kyphosis correction obtained during first surgery growing rods, through cantilever manoeuvre, seem to grant a better sagittal plane restoration compared to VEPTR.

**Figure 1:**
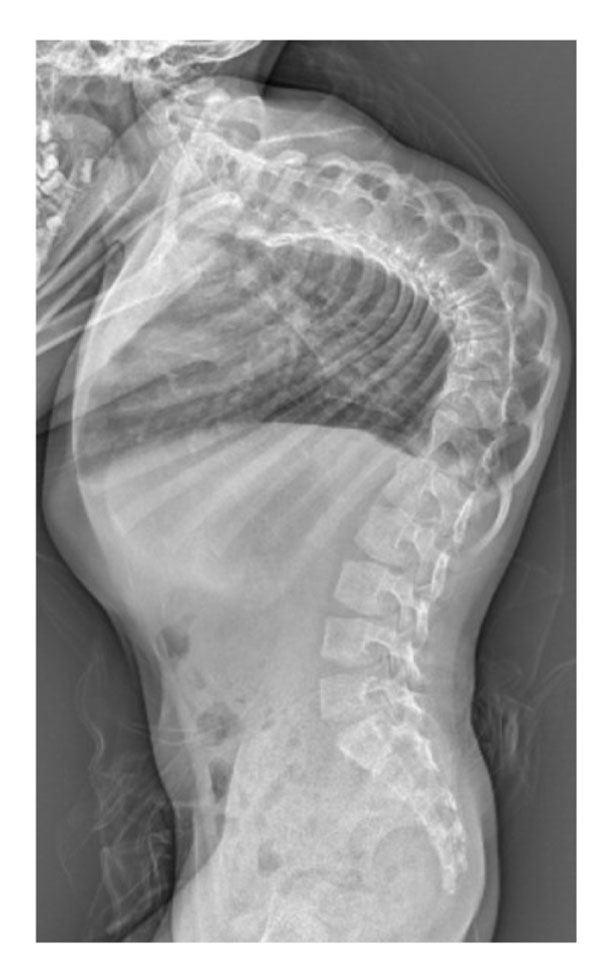


**Figure 2:**